# From Sensor to Observation Web with Environmental Enablers in the Future Internet

**DOI:** 10.3390/s110403874

**Published:** 2011-03-30

**Authors:** Denis Havlik, Sven Schade, Zoheir A. Sabeur, Paolo Mazzetti, Kym Watson, Arne J. Berre, Jose Lorenzo Mon

**Affiliations:** 1 AIT–Austrian Institute of Technology GmbH, Donau-City-Strasse 1, A1220 Vienna, Austria; 2 Institute for Environment and Sustainability, European Commission, Joint Research Centre/TP 262, Via E. Fermi, 2749, I-21027 Ispra (VA), Italy; E-Mail: sven.schade@jrc.ec.europa.eu; 3 University of Southampton IT Innovation Centre, 2 Venture Road, Chillworth, Southampton, SO16 7NP, UK; E-Mail: zas@it-innovation.soton.ac.uk; 4 Institute of Methodology for Environmental Analysis, CNR–National Research Council of Italy, 85050 Tito Scalo (PZ), Italy; E-Mail: mazzetti@imaa.cnr.it; 5 Fraunhofer Institute of Optronics, System Technologies and Image Exploitation, 76131 Karlsruhe, Germany; E-Mail: kym.watson@iosb.fraunhofer.de; 6 SINTEF-Foundation for Scientific and Industrial Research, Norwegian Institute of Technology, N-0317 Oslo, Norway; E-Mail: arne.j.berre@sintef.no; 7 ATOS Origin S.A.E., 28037 Madrid, Spain; E-Mail: jose.lorenzo@atosresearch.eu

**Keywords:** future internet, environmental usage area, sensor web, observation web, requirements analysis, environmental enablers, open standards, internet of services, internet of things, internet of content, internet of people

## Abstract

This paper outlines the grand challenges in global sustainability research and the objectives of the FP7 Future Internet PPP program within the Digital Agenda for Europe. Large user communities are generating significant amounts of valuable environmental observations at local and regional scales using the devices and services of the Future Internet. These communities’ environmental observations represent a wealth of information which is currently hardly used or used only in isolation and therefore in need of integration with other information sources. Indeed, this very integration will lead to a paradigm shift from a mere Sensor Web to an Observation Web with semantically enriched content emanating from sensors, environmental simulations and citizens. The paper also describes the research challenges to realize the Observation Web and the associated environmental enablers for the Future Internet. Such an environmental enabler could for instance be an electronic sensing device, a web-service application, or even a social networking group affording or facilitating the capability of the Future Internet applications to consume, produce, and use environmental observations in cross-domain applications. The term “envirofied” Future Internet is coined to describe this overall target that forms a cornerstone of work in the Environmental Usage Area within the Future Internet PPP program. Relevant trends described in the paper are the usage of ubiquitous sensors (anywhere), the provision and generation of information by citizens, and the convergence of real and virtual realities to convey understanding of environmental observations. The paper addresses the technical challenges in the Environmental Usage Area and the need for designing multi-style service oriented architecture. Key topics are the mapping of requirements to capabilities, providing scalability and robustness with implementing context aware information retrieval. Another essential research topic is handling data fusion and model based computation, and the related propagation of information uncertainty. Approaches to security, standardization and harmonization, all essential for sustainable solutions, are summarized from the perspective of the Environmental Usage Area. The paper concludes with an overview of emerging, high impact applications in the environmental areas concerning land ecosystems (biodiversity), air quality (atmospheric conditions) and water ecosystems (marine asset management).

## 1. Introduction

In 1999, Cherry Murray pronounced a vision of the World’s “electronic skin” [1]:

“In the next century, planet Earth will don an electronic skin. It will use the Internet as a scaffold to support and transmit its sensations. This skin is already being stitched together. It consists of millions of embedded electronic measuring devices: thermostats, pressure gauges, pollution detectors, cameras, microphones, glucose sensors, EKGs, electroencephalographs. These will probe and monitor cities and endangered species, the atmosphere, our ships, highways and fleets of trucks, our conversations, our bodies—even our dreams.”

One decade after Murrays’ statement, we are now witnessing a tremendous development of low-cost miniaturized sensors and wireless sensor nodes. The Web 2.0 has evolved from a fringe curiosity to a mass phenomenon, and the Sensor Web Enablement standards suite of the Open Geospatial Consortium (OGC SWE) [2] provided the framework to develop an “Observation Web” with observations originating from humans, sensors or numerical simulations. The rapid development and pervasive deployment of mobile communication devices, computers and sensors that are connected to the Internet (Web), together with a growing number of application services on the Web, is driving the advancement of the Future Internet. At the same time, society is becoming increasingly aware of the need for sustainable, smart and inclusive growth with full protection of the environment and quality of life. Nevertheless, most Web 2.0 applications remain unaware of the state of the environment. At the same time, the environmental applications do not use the decentralized and collaborative nature of today’s Internet to its maximal extent. Consequently, the actual exploitation of the environmental observations and systems lags behind the increasing demands for timely and contextually aware information delivery.

This paper provides an overview of today’s societal and technical challenges that are required for the implementation of the next generation Earth Observation (EO) infrastructures applications connecting users’ socio-economic interests with environmental observations. We pay special attention to the European “Future Internet” initiative as the fundamental Information and Communication Technology (ICT) platform to be put in place for achieving such challenges in this new decade. However, the ideas presented in the paper are believed to equally valid for complementary research activities, such as the US-led “Internet2” [3]. The remainder of this paper is organized as follows:

1. The first section summarizes the scientific context of the presented work. We introduce the concept of the Future Internet, argue for the importance of the Environmental Usage Area, and provide an overview of the “envirofied” Internet of the future.
2. Section 2 presents some recent ICT trends relevant to the Environmental Usage Area. This provides a first look at the challenges and opportunities, which are likely to occur.
3. Section 3 provides a more detailed analysis of the technical challenges within the Environmental Usage Area of the Future Internet. This section also includes pointers to potential “environmental enablers” for the Future Internet.
4. In Section 4, we discuss the arising challenges and opportunities on the base of three application scenarios with high scientific and societal potential.
5. Our conclusions and an outlook to future work are presented in the last section (Section 5).

### 1.1. ICSU Grand Challenges in Global Sustainability Research

The understanding and management of climate change, environmental degradation, and the quest for sustainable growth are among the most fundamental challenges facing humanity in the 21st century. This priority is articulated by the International Council for Science (ICSU) as follows [4]:

“Over the next decade the global scientific community must take on the challenge of delivering to society the knowledge and information necessary to assess the risks humanity is facing from global change and to understand how society can effectively mitigate dangerous changes and cope with the change that we cannot manage. We refer to this field as ‘global sustainability research’. Global sustainability research provides a new holistic approach to science, building upon and integrating expertise within the sciences (social, natural, health, and engineering) and humanities. This holistic vision will contribute to provide innovative responses to the pressing coupled social-environmental research questions of human interactions with the Earth system.”

The ICSU argued that two main shifts need to take place in the world of science in order to develop research on global sustainability:

- There needs to be a much greater collaboration and integration between the natural and social sciences, health sciences and engineering to integrate the complex inter-relationships between physical and socio-economic processes effectively.
- The increased emphasis on multi-disciplinary research must be coupled by a much greater involvement in the research process of external stakeholders through an open and participatory approach, and building trust in science while encouraging all actors to take responsibility with their collective mitigations and adaptations to change.

**Figure 1.**
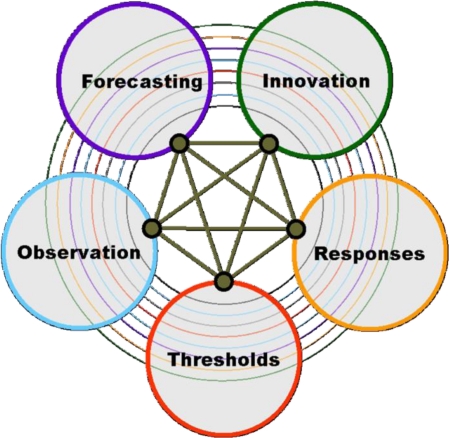
The five ICSU identified Grand Challenges in Global Sustainability Research as identified by ICSU.

Following a broad consultation involving over 1,000 scientists from 85 countries during 2009–2010, ICSU identified five scientific priorities, or Grand Challenges, in global sustainability research (Figure 1).

These Grand Challenges include the following:

1. Developing the observation systems needed to manage global and regional environmental change.
2. Improving the usefulness of forecasts of future environmental conditions and their consequences for people.
3. Recognizing key thresholds or non-linear changes to improve our ability to anticipate, recognize, avoid and adapt to abrupt global environmental change.
4. Determine what institutional, economic and behavioural responses can enable effective steps toward global sustainability.
5. Encouraging innovation (coupled with sound mechanisms for evaluation) in developing technological, policy, and social responses to achieve global sustainability.

### 1.2. Future Internet and Europe 2020 Strategy

In response to the unprecedented world economic crisis in 2008, the European Commission formulated its long term vision in Europe 2020 strategy [5] which emphasizes actions around three main priorities: (i) smart growth: developing an economy based on knowledge and innovation, (ii) sustainable growth: promoting a more resource efficient, greener and more competitive economy, and (iii) inclusive growth: fostering a high-employment economy delivering social and territorial cohesion.

**Figure 2.**
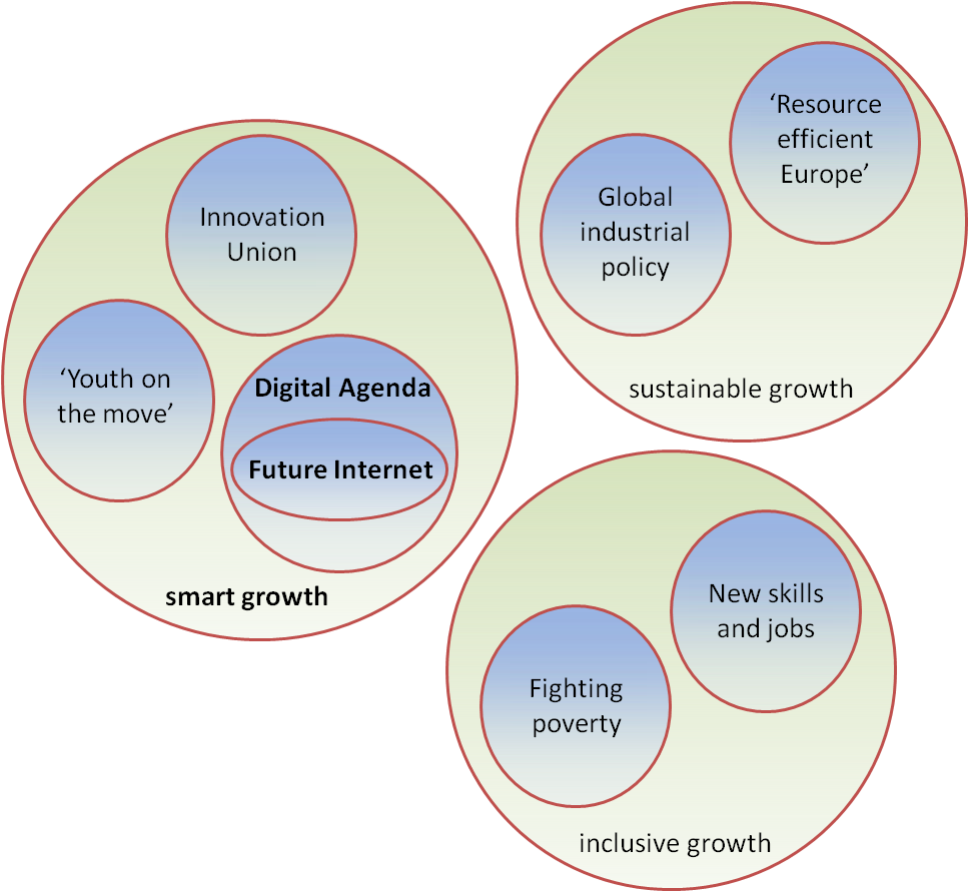
Future Internet and the Europe 2020 strategy.

Seven flagship initiatives give substance to the strategy. They specifically address: innovation, youth and the labour market, digital agenda, resource efficiency, industrial policy in the global context, skills and jobs, and social and territorial cohesion respectively. In particular, the Innovation Union [6] initiative aims to improve conditions and access to funding for research and innovation in Europe, to ensure that innovative ideas can be turned into commercial products and services that create jobs and economic growth, whereas the Digital Agenda for Europe [7] outlines policies and actions to maximize the benefit of the Digital Revolution for all. The Digital Agenda foresees the action to “*work with the Member States and stakeholders to implement cross-border eEnvironment services, notably advanced sensor networks”* under the eGovenment theme. Future Internet [8] is part of the Digital Agenda for Europe’s efforts to deliver economic benefits from fast to ultrafast Internet and interoperable applications. The overall situation is depicted in Figure 2.

**Figure 3.**
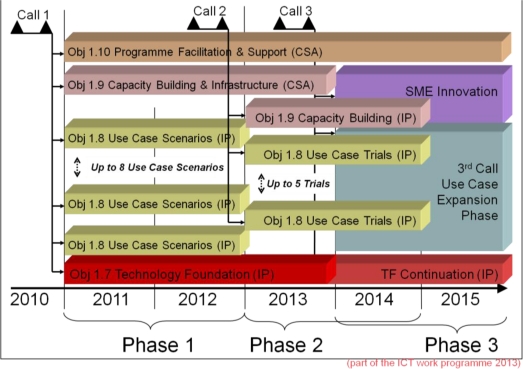
Timeline of the future internet public-private partnership programme (from Europe’s Information Society Thematic Portal).

The European Commission (EC) provided €90 million for funding Future Internet-related research in 2011, and a further €210 million in 2012–2013 through the “Future Internet Public Private Partnership” (FI-PPP) FP7 Programme. The FI-PPP aims to: (i) to support an Internet-enabled service economy, (ii) to improve key ICT infrastructures of Europe’s economy and society by making them better able to process massive amounts of data originating from multiple sources; (iii) to render the Internet more reliable and secure; and (iv) to allow real time information to be processed into real time services. In order to maximize the effects of Research and Development (R&D) Investments, the FI-PPP calls for proposals feature two important requirements. They are:

1. The FI-PPP programme foresees three distinct development phases, each corresponding to one FI-PPP call (Figure 3): phase one looks at integrating the underlying technology and developing use case scenarios; phase two looks at making available the Future Internet core platform, large scale trials and pilots; and phase three massively broadens the scope to large-scale trials with real applications.
2. The FI-PPP calls demand strong cross-project coordination, knowledge exchange, and free re-use of results across all FI-PPP projects. This is in sharp contrast with the usual EC research programmes, and clearly indicates the strong political will to make Europe a world leader in Future Internet research and roll-out of future innovative Internet technologies needed to “smarten up” infrastructures in areas affecting humans daily lives such as health, transport and energy.

### 1.3. Societal Importance of the ICT for Environment

Both the ICSU Grand Challenges and the Digital Agenda for Europe explicitly acknowledge the societal importance of ICT for environment as one of the key enablers for sustainable development and improved quality of life. *Most Europeans value a healthy environment, and over 60% agree that policies aimed at protecting the environment are a motivation to innovate. Yet, 42% feel badly informed with* the *greatest lack of information on* the *impacts of environmental change* [9]. The Digital Agenda therefore identifies ICT for the environment as a critical area to deliver environmental, social and innovation-led growth objectives. In this vision, the opportunities of new technologies such as Sensor Web, and smart grids play a particularly important role.

The European commitment to sustainable growth and innovation through investment in ICT for the environment is demonstrated by the development of the Infrastructure for Spatial Information in Europe (INSPIRE), the transition from research to operations of the Global Monitoring for Environment and Security (GMES) initiative, the development of the a Shared Environmental Information System (SEIS), and the combination of all three as a European contribution to the Global Earth Observation System of Systems (GEOSS) initiative, which recognizes the global nature of environmental problems.

**Figure 4.**
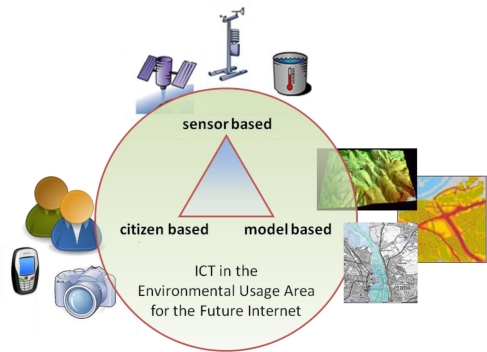
Technology triangle: observations from sensors, humans and models.

Given the policy and scientific settings outlined above, there is a clear opportunity to further develop the threefold sustainability-innovation-growth relationship by dedicating a usage area of the FI-PPP to the environment. This paper elaborates this need and particularly focuses on the opportunities provided by new technologies to improve the quality and timeliness of Observations of the environment since these are the building block which underpin the other four Grand Challenges (Figure 1). In particular, we address the technology triangle that was identified in the “Towards a Future Internet Public Private Partnership: Second Usage Area Workshop” in Brussels, on 21st and 22nd June 2010 (Figure 4; [10]).

“Sensor based” technology just achieved a major breakthrough as physical sensors became Web enabled; “citizen based” technology, also known as Volunteered Geographic Information (VGI), faces a new area of mass deployment; and “model based” technologies provide major interoperability challenges. Not surprisingly, the Sensor Web, or better the Observation Web is considered as the “next big thing” in the development of smart systems [11].

The reason why we propose the term Observation Web rather than the more commonly used Sensor Web in this paper is to emphasise on the fact that the OGC defines the term Observation as a piece of structured and semantically very rich information containing for example the value, unit, temporal-, domain- and spatial-context, provenance, ownership, quality, uncertainty and process description. Observations may indeed be a result of “direct” observation of the natural world by people or sensors, but the OGC definition explicitly includes the idea of historic observation repositories, and observations generated by various numerical models. The advantages of harmonizing the concepts of observations independently from their origins were clearly demonstrated in the SANY project [12,13].

### 1.4. Environmental Usage Area for the Future Internet

A great deal of effort has already been invested in improving interoperability of data across both environmental domains and administrative borders within the areas of “ICT for Environment”. Environmental informatics initiatives have already achieved great progress in some areas related to interoperability of services (applying the Model Web principles [14]), context aware intelligent data management, mining and fusion services, as well as estimation, communication, and propagation of uncertainty along the added value chain of environmental information. In addition, huge progress has been achieved at the level of ad-hoc sensor networks. However, more work still needs to be done on the harmonization of environmental data within and across domains, especially with respect to biotic observations, and more importantly when dealing with large heterogeneous data holdings. Much of the existing work included workarounds for shortcomings of the current Internet infrastructure, in particular with respect to robustness, scalability, and quality of service. The amount of observation data, its growing volume and heterogeneity thus still has to be addressed across geographic scales and the reliable and timely discovery and delivery of environmental information still has to be matched with the EU’s targets and objectives. Related research has already triggered innovation, but has yet to be leveraged to meet challenges on European level, which provides a dynamic on-request understanding of the Earth’s atmospheric, marine and terrestrial spheres for the benefit of all European citizens.

With the FI-PPP initiative aiming to solve some of the key issues encountered by ICT for Environment, it is very important to protect existing investments in environmental data provision to the full extent, while easing the application development and data provision by non-experts in the future. Ideally, the entire environmental infrastructure should be seamlessly integrated into the Future Internet applications, and resulting into:

1. Environmentally enabled (“envirofied”) Future Internet with context-sensitive applications; making extensive and transparent use of the environmental observations; and
2. Future Internet enhanced Environmental ICT infrastructure; making use of security, reliability, capacity, throughput and scalability inherent to the Future Internet.

Relevant information sources include real-time geo-referenced sensor observations, maps, geo-referenced data repositories and archives with semi-static and geo-localized character, numerical simulation and forecast of environmental processes, subjective and objective user-contributed information in various forms (for example human language text such as Twitter and Facebook entries, social opinions, messaging and video scenes of environmental events of interest), and other semi-structured information sources, such as environmental consultancy reporting and scientific publications.

**Figure 5.**
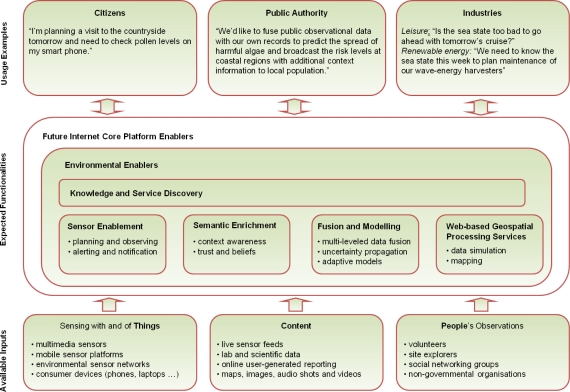
Overview of the envirofied Future Internet and its collaborating stakeholder groups.

Environmental enablers for the Future Internet shall provide the data access and knowledge management infrastructure of the environmental service space. They shall federate existing (open standard based) technologies, as well as different architectural styles in small-scale pilots and trials, and at the same time facilitate large-scale applications in terms of geographic extents, data volumes, and processing capacity. The addressed components involve various kinds of things, services, content, and people (Figure 5).

The sensing (observations) of natural and man-made things within our environment provide added-value data processing to the Future Internet platform. These observations can originate from many sources including: large monitoring systems; individual citizens, sensors embedded in ubiquitous items such as cars and cellular phones; environmental simulation models; as well as from secondary information sources (such as cadastres).

The “Environmental Usage Area” for the Future Internet will allow the required components to discover, access and process information related to the environment. The required functionalities need to be offered in such a way that a broad range of different users can benefit from them (Figure 5).

## 2. Recent ICT Trends in the Environmental Usage Area

Previous section summarized the scientific context and illustrated the societal importance of the proposed work. In this second section, we present several recent ICT trends that are of high relevance to the future Environmental Usage Area, but hardly used at all in state of the art environmental applications. This provides a first look at the challenges and opportunities, which are likely to occur, and prepares the stage for the more detailed analysis in the Section 3.

The phenomenal growth in the usage of the Internet, linking millions of people through computers, social networking and mobile communication devices, together with the rapid development of affordable smart sensors in the last few years are expected to have profound effects on the way environmental observations are generated and accessed by communities in the future. The resulting large scale interconnections of these objects shall genuinely lead to a paradigm shift in the way the Future Internet enables the social communities to understand how changing environment could affect their living, and also how they can adapt their lives to the changes occurring in their environment at more localized scales. Large communities shall be able to monitor the natural environment and share such information with other users of the Future Internet, as well as to use this data in applications outside the environmental domain. We present illustrative trends towards such envirofied Internet below.

### 2.1. Ubiquitous Sensors and Opportunistic Sensing

The quality of miniature low-cost sensors is steadily improving; their prices are falling and mass deployment becoming a reality. In modern cars, one can monitor air and road temperature, as well as COx and NOx concentrations at the combustion engine using built-in sensors. Also, with current mobile phones one can perform audio and video recording of the environment using in-built microphone and video cameras. Most of these mobile phones are also equipped with a GPS position sensor, accelerometer and compass. New sensor development for mobile phone include miniaturized pressure sensors, dual-microphone solutions for ambient noise cancellation, and more specialised air quality sensors [15]. Another important technological development is the usage of energy autarkic sensor platforms with wireless ad-hoc networking capabilities. These platforms scavenge the energy required for powering sensors, their processing and communication units from their ambient environment, and no longer require any external cable based power supply or change of batteries. Depending on the ambient environment conditions, the autarky may be achieved by scavenging power from human physical motions, ambient heat, light, radiation, wind, or waves [16].

The relatively low prices of these devices allow their mass-deployments for environmental monitoring. Nevertheless, their relatively modest quality of measurements when compared to state of the art environmental monitoring systems means that they can only complement them, particularly in providing new spatially localised environmental observations. This consequently brings us to address the concept of “Opportunistic Sensing”. This concept means exploiting various types of sensors for environmental monitoring tasks for which they were not initially designed. For example, the built in gas sensors in automotive vehicles could potentially be used as a proxy for the measurement of traffic-induced emissions. Further, cellular phone microphone(s) can be used for sensing ambient noise levels [17] and the accelerometers in PCs and smart phones for sensing seismic events [18].

When compared to environmental monitoring systems, opportunistic sensing provides low cost high spatial and temporal coverage of environmental observations. However, the use of opportunistic sensing in real world applications is hampered by many challenging factors. Some of these are summarised below:

1. Available sensors are generally of low quality when compared to those used in traditional environmental monitoring networks;
2. The availability of sensor data at a particular point in space and time cannot be guaranteed;
3. Standard quality assurance in sensor measurement, including sensor calibration by sensor owners cannot be enforced.

The inexpensive and simple use of Internet connections, sensors, and applications is currently empowering citizens to organise community-run observation projects. Two main models for sustainable citizens’ managed observation networks are emerging in this decade. These include: (a) The Citizen Scientists, who collect and share observations as part of their recreational hobbies or vocational interests; and (b) the “grassroots observation networks” providing supporting evidence on environmental processes for citizens and community groups.

One of the first citizens-run observation networks providing evidence against industrial operators was the Dutch “Geluidsnet”. Geluidsnet started in 2003, as part of a citizen’s pressure group who wanted to prove correlations between generated urban noise above accepted regulated standards with local air traffic operations [19]:

“Geluidsnet has installed a large number of strategically placed unmanned noise meters which continuously transmit their measurements to a central database using Internet connections. The meters themselves are compact, low-cost and low-maintenance, and can be positioned in almost any location at ground or roof levels. They operate continuously, and transmit their results over the Internet to a central database. Geluidsnet is thus not limited by the high cost of human resources or equipment, in contrast to more traditional methodologies deployed for environmental monitoring.”

As a result, Geluidsnet widely overcame the expectations of many communities since it turned into a commercial organization, specializing in noise pollution monitoring using multiple subsidiary deployments in the Netherlands.

### 2.2. Crowd-Sourcing and Human-Centred Sensing

**Figure 6.**
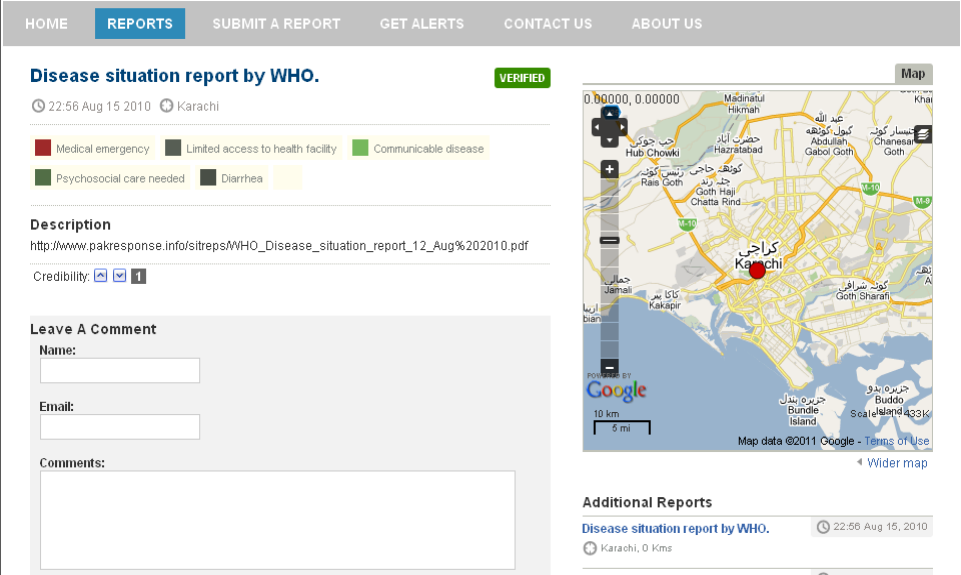
Ushahidi report illustration, from Pakrelief web site [22].

A different approach to VGI is embraced by Ushahidi [20]. Rather than concentrating on the sensors, the Ushahidi team developed a technology that simplifies volunteering unstructured geo-referenced information through a variety of channels (e-mail, SMS, web …). The so called Crowd Sourcing is complementary to grassroots sensing initiatives such as Geluidsnet, in a sense that it allows citizens to “record (their observations of) an event as it is happening” rather than relying on scarce and possibly censored official reports. The recent earthquake in Haiti has clearly shown the value of the Ushahidi approach as an observation and communication platform for crisis event observations [21]. Figure 6 illustrates both the features and some of the shortcomings of the Ushahidi reports.

In addition to title, textual description, and optional photography, each report is associated with its author, reporting time, location, and one or more categories. Furthermore, the report has several quality-assurance related features: (1) each report is automatically linked with additional reports from the same area, as well as with the additional reports from the same author; (2) other users can both add their own comments to the report as well as indicate their opinion on report’s credibility; and finally (3) the administrators can mark the report as “verified”. While these features certainly help improving the quality and usability of reports, the platform does not appear to track the overall credibility of the users, and the comments carry no a-priory semantics. Moreover, the Ushahidi reports are not well suited for automated processing: the main information content is in an unstructured form, and the geo-temporal context information is limited to a single point in time and space. In the case of the report shown above, the actual information is in an external PDF file, and the analysis of this file reveals that the area of relevance is the whole flooded area in Pakistan, and the relevant time frame is from 31 July to 12 August 2010 [22].

A different approach to crowd sourcing is used by eBird application [23]. It allows the citizen scientists to enter precise data on bird sightings and explore the generated database. The data collected by eBird is both specialized and highly structured, with main data load consisting of pre-defined species names, counts of individual birds for each of the recorded species, area of interest and the time span in which the sighting was performed.

So far, no generic applications for structured VGI collection, processing and visualization exist. However, the OGC SWE can potentially provide much of the needed functionalities. Specifically, if we consider VGI as “observations by humans”, it is possible to describe the observation process using Sensor Model Language (SensorML) and encode the actual data using the Observation and Measurement Model (O&M). The querying capabilities of the Sensor Observation Service (SOS) are at least on-par with those offered by eBird or Ushahidi, and the transactional SOS interface provides a standardized way to define new observation types and write the observations to the underlying SOS database. Unfortunately no applications capable of automatically generating the appropriate graphical user interfaces for each type of observations defined in the underlying SOS have been developed. In addition, the development of distributed semantic tagging may help us to improve both the semantic content and the quality of reports [24].

Harnessing of opportunistic sensing observations, including those from direct human sensing bring several new challenges in addition to their large volume and quality assurance related issues. These challenges may include:

1. Human sensing observations are inherently subjective.
2. The sensitivity of human sensing observations on an environmental process can vary widely, for example this may depend on a person state of awareness and alertness.
3. Human sensing reliability inconsistencies. Humans are usually inefficient in maintaining good quality in repetitive tasking. They may completely fail to report and mis-interpret environmental, observations.

The most promising area for the use of Human-Centred Sensing is the notification of unusual environmental events which can be easily quantified and described as a step function pattern. For example, most human observers can easily distinguish between “sunny weather” *versus* “cloudy”, “calm” *versus* “windy”; or “dry” *versus* “wet” or “snow”. It is nevertheless unexpected that citizen scientists will report every rain episode, although they may be indeed eager to report the “first snowfall”. Even more interestingly is the human’s ability to provide more intelligent observations of some environmental events that are otherwise quite difficult to sense automatically. These may include pattern-recognition type of observations (such as “the swallows are back”, “the river colour changed yesterday” or “ambrosia plants are growing on this field”). Nevertheless, the authors acknowledge that a lot of research in computer vision has been conducted over the years in the development of automated pattern recognitions based monitoring systems.

### 2.3. Converging Realities

The Future Internet will increasingly be used as an additional mean to support our natural senses while we observe and recognise patterns in our environment with greater contextual awareness. In addition to already established annotated and colour-coded interactive maps, the Future Internet shall be marked by Virtual Reality, Augmented Reality, and possibly even Real Virtuality [25,26] applications.

Virtual reality based applications come in two main forms. First, there are applications which allow users to navigate into a virtual world without performing any physical motion. The user’s view of the virtual environment can be from the avatars perspective (“first person”) or from the third person perspective. Some applications, including the famous “second life” [27] allow the users to explore computer generated spaces and interact with other users and things within these spaces without physical motions.

Real Virtuality applications go even further in their attempt to completely immerse the users into the virtual reality world. With the help of special equipment, Real Virtuality applications provide information for all five human senses delivered in a natural manner.

**Figure 7.**
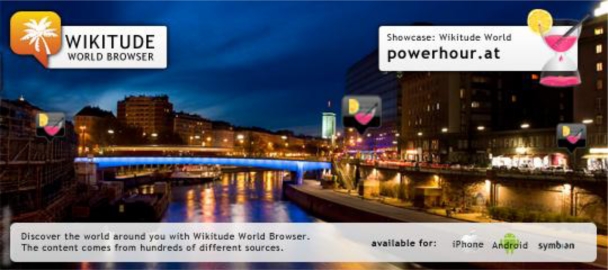
Wikitude World Browser illustration (from Wikitude web site [28]).

Finally, Augmented Reality applications superpose the additional information onto the real world (for example seen through cellular phone camera or through special augmented reality glasses). Thanks to the development of smart phones, augmented reality applications have penetrated the everyday life. Unfortunately, the mainstream applications such as Wikitude World Browser (Figure 7; [28]) operate with informative tags associated with a single Point of Interest (POI), and therefore currently cannot be easily used to represent environmental information.

**Figure 8.**
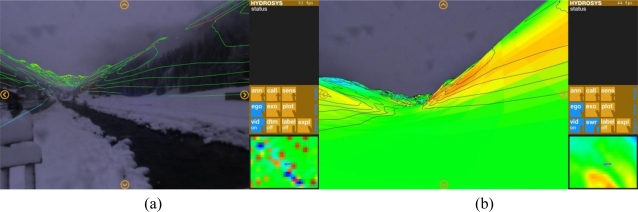
HYDROSIS augmented reality application prototype superposing environmental information on a camera picture as (**a**) iso-lines, and (**b**) colour-coded areas. Courtesy of Eduardo E. Veas (veas@icg.tugraz.at).

The HYDROSYS project team recently proposed an enhanced version of an augmented reality application for the environmental domain usage (Figure 8; [29]).

An alternative visualization technique, using coloured spheres of varying sizes to represent environmental information has been demonstrated by the SiteLens team [30]. Unlike Wikitude and other established applications operating with points, HYDROSIS and SiteLens applications shall be capable of superposing environmental status information in form of isotopes and colour-coded areas.

Since the use of augmented reality applications requires knowledge of the users’ position and orientation in the real world, and virtual reality applications do similarly for a user’s avatar in the virtual world, it is possible to merge the two paradigms and allow interaction between users who participate in the same application.

Even the relatively simple Converging Reality applications could in many cases allow sharing of information and teamwork with only part of the team present in a certain location. Some immediate usage of this type of applications may include leisure and educational applications, such as the guided museum tours in a “virtual museum”, or near-reality tours around historic scenes and buildings—accessible through virtual reality for the remote participants and superposed to today’s scenery for visitors of the original location;

Further functionality can be achieved through the integration of various environmental observations, model results and elements of the “Internet of Things” in convergent reality applications. Eventually, the environmental information superposed on real and imaginary 3D objects shall allow users access to unprecedented insights about status, history and outlook of their surroundings with individual objects (things, plants, animals).

## 3. Technical Challenges of the Environmental Usage Area

The trends which have been outlined above indicate the changes ICT for environment will undergo in the near future. They also provide a first look at the challenges and opportunities, which are likely to occur. This section provides a more detailed analysis of the technical challenges that have so far prevented the “envirofication” of the Internet and subsequently present the emerging solutions for the following issues:

1. Variety of requirements on observation gathering, events identifications, processing and presentation that cannot be met by the existing Service Oriented Architecture (SOA) designs [31,32];
2. Scalability and reliability of the infrastructure that is running on top of essentially unreliable Internet infrastructure;
3. Various security related issues, including the authentication, authorization, access control, confidentiality, integrity, non-repudiation, auditing, privacy and trust;
4. Overcoming the semantic heterogeneities, multiple formats, accuracies and degrees of beliefs in data sources and allowing the process of intelligent context-aware information retrieval;
5. Making sense of large sets generated from heterogeneous observations and multiple sources;
6. Providing individual experimentations and deployment environments that allow simultaneous use of various observations sources, services, things, people and other content in envirofied applications;
7. Assuring on-demand and automated delivery of context aware information to a diverse community of environmental stakeholder groups with different requirements.

In the following section (Section 4), we will take a more extensive look at these opportunities by sketching scenarios for three environmental domains, in which we expect high scientific and socio-economic impact.

### 3.1. Designing a Multi-Style Service Oriented Architecture

Service-oriented architecture (SOA) is widely accepted as the paradigm of choice to loosely couple software components in distributed applications. However, no agreed conceptual foundation of a geospatial SOA, that is an agreed service meta-model that is also compliant with geospatial service standards of OGC currently exists [33] In fact, a number of competing architectural paradigms evolve in-parallel, each with their own respective advantages in certain usage areas. It is therefore important to investigate the possibilities to harmonize existing and emerging service meta-models and ontologies of OMG (SoaML) or OASIS (SOA reference models) with ISO/OGC (ISO 19119 [34], OGC 07-097 RM-OA [35]), as well as with the elements from autonomous agents [36,37], Resource Oriented-, and Event Driven-Architecture (EDA) [38] with the objective of conceptualizing a so-called multi-style SOA.

**Figure 9.**
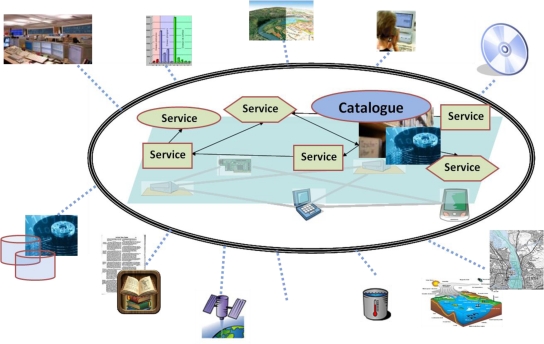
Service infrastructure (from RM-OA [35], slightly adapted).

A multi-style SOA is a SOA that supports multiple architectural styles and communication patterns such a request/reply messaging, event-driven interactions and resource-oriented services (commonly known as RESTful Web services) following a unified service meta-model [33]. Multi-style SOA for Environmental Usage Area of the Future Internet should be rigorously based upon comprehensive SOA design patterns [39] and leverage the Future Internet functions. The flexibility offered by multi-style SOA shall allow us to build mash-up applications for multiple usage scenarios described in Section 4, with large datasets, VGI, and “plug-and-observe” applications of environmental sensors and models. A schematic diagram for a SOA as the service infrastructure for multiple applications and users is shown in Figure 9 below.

Independently of the architectural style, the future architecture shall support the following the generic functionalities:

1. Semantic annotation of the SOA allowing bridging multiple thematic communities and enabling cross-domain applications;
2. Composition of new services from existing ones, through service orchestrations. Service Orchestration greatly speeds up development of the new services and environmental applications.

The specification of a concrete architecture, components, interfaces, and other characteristics of a Future Internet service infrastructure for environment requires careful design. Design is the process of defining the architecture, components, interfaces, and other characteristics of a system or component as well as the result of this process. In order to fulfill the requirements of a user who is expert in the thematic domain, there is a need to coordinate his/her actions with the system designer [40].

On the one side, the user transforms the problem into a set of user requirements that represent expectations about the functionality and the characteristics of the resulting system. On the other side, the system designer interprets the user requirements into a software specification. Hence a common semantic understanding of the information elements across the system functions is important to achieve, while information requirements should be treated explicitly on the same level as the functional and non-functional requirements [33]. As a result, requirements can be categorised as follows:

1. Functional requirements that describe functions and processes,
2. Informational requirements that describe the major terms, concepts of the application domain and information elements the system needs to process, and
3. Non-functional requirements. These can be subdivided into two categories:
  3.1 Qualitative requirements such as dependability and security aspects, and
  3.2 Side conditions that describe design constraints, for instance, the request to use standards, or to leverage emerging capabilities of the Future Internet.

**Figure 10.**
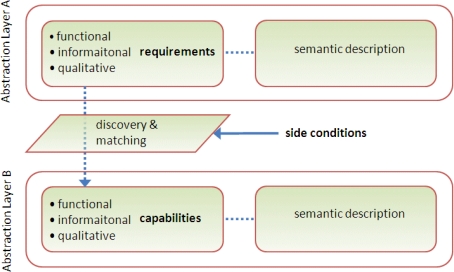
Mapping of requirements to capabilities (from [33]).

The key challenge for a service-oriented design is semantic matching of requirements at one abstraction layer (A) to capabilities of another abstraction layer; and (B) taking side conditions into account (Figure 10).

Discovery and matching, for example using the geospatial service catalogues defined by ISO and OGC, require an associated semantic description of both requirements and capabilities in order to be effective. Such semantic descriptions give meanings to the terms used in the specifications.

The combination of the tasks of discovery and matching plays a key role in SOA design. It serves as a generic mechanism to bridge the gap between heterogeneous descriptions and/or expectations. This kernel problem occurs repeatedly when user requirements are broken down into multiple steps across several abstraction layers. In fact, capabilities turn into requirements for the next design step [33], leading to a stepwise and iterative co-development of requirements and architectural artefacts [41]. These steps are summarised below.

1. In the Analysis step, the user analyses the problem and specifies user requirements.
2. In the Abstract Design step, the user requirements are matched with the capabilities of an abstract service platform, for example through a service model of the multi-style SOA.
3. In the Concrete Design step, the capabilities of the abstract service platform turn into requirements for the design of the concrete service platform (here: the Future Internet platform) and finally result in the specification of the platform capabilities.
4. In the Engineering step, the specified capabilities of the concrete service platform are implemented as service components (environmental enablers) and deployed in the context of the concrete (Future Internet) platform.

### 3.2. Scalability and Robustness of Environmental Service Networks

Environmentally enabled applications often require (near) real time processing of large amounts of data. One of the primary objectives of envirofied Future Internet is therefore to establish both scalable and robust networks of services which secure continuous operability and assured in-time delivery of data and information. It is absolutely paramount to achieve evolving infrastructures with autonomous, adaptable, and to a high degree, configurable service components. A Multi-Style Service Oriented Architecture as proposed in the earlier section shall adopt these criteria efficiently. Functional integration on the basis of standardized services and a multi-style SOA shall enable the following capabilities:

1. Seamless “plug-and-measure” type operation for new sensors, sensor networks and various processing services. This is a key element for enabling various communities to conduct measurement campaigns and explore observations from proprietary sensors using standardized client applications.
2. Homogenized handling of the services at different levels of details that span from the environmental observations to downstream structured data fusion, uncertainty propagations and feedback with intelligent learning.
3. Structured and multi-levelled data fusion services capable of overcoming sensor breakdown events while providing continuous information feeds to downstream services for various communities.
4. Finally, the future applications shall require in-network processing: a networked cluster of sensors shall provide a consolidated answer to an environmental question without relying on a central processing instance. This is essential to control the amount of data communicated across networks, especially in highly dynamic ad-hoc scenarios with mobile sensors, sensors with limited energy supplies, multiple publishers and consumers (clients) of the same or related information.

Unlike scalability, which can be adequately handled at the application level, the robustness of the future envirofied applications could be greatly improved at the lower levels of the Internet stack. Indeed, an expensive dedicated network infrastructure is the only way to assure timely delivery of information over the Internet today. In order to overcome this issue, the Future Internet infrastructure should provide means to assure the minimal level of services for priority applications even in the emergency situations. Possible ways to achieve this goal are, for example smart switches capable of assuring the contractual level of service for priority customers and the packet-level prioritization.

### 3.3. Intelligent Context-Aware Information Retrieval

The context-aware and on demand delivery of information relevant to the users (citizens, organizations, groups, *etc.*) of envirofied applications is key for the necessary queries or actions they want to pursue regarding environmental observations.

Users’ context consists of their present, previous and predicted profiles, queries or actions. Their actions for example could refer to environmental observations contributed or annotated by the user. Context can therefore include activity information, event history, spatial and temporal information, resources the users have access to and the group of people with which they are collaborating. The central task of context-aware retrieval is to match the context of the users’ search with the context of available information elements. Key concepts in this field may include the user’s query, a query context, and a collection of distributed information sources and sets of individual documents from these sources, possibly broken down into fields, from these sources. Queries can be interactive, pro-active or continuous. Information extraction technology [42], as well as annotations contributed by users may be employed to enrich metadata within textual documents.

At the heart of a context-aware retrieval engine is a matching algorithm that takes the user’s context together with a potential document to be retrieved and score it on relevance. Some of the matching algorithms which can be used in envirofied applications include topic tracking [43], fuzzy matching [44], and context aware caching [45].

Environmental data sets are continuously growing, and many use cases critically depend on the availability of the latest data sets. The envirofied information retrieval facilities therefore need to assure the ongoing real-time indexing of the large and ever-growing environmental data sets, for example through use of the real-time distributed indexing techniques and matching strategies.

### 3.4. Data Fusion, Modelling and Uncertainty Propagation

In recent years, low level reusable data fusion services have been successfully deployed in a SOA context (see also Section 3.1) to support multiple environmental domain web-service applications [46,47]. Nevertheless, the deployment of higher level fusion services for environmental applications in a SOA context remains challenging. Higher level fusion services [48] are currently seen as one of the key Future Internet enablers to be deployed for future web-based applications in the environment usage area and beyond. This new generation of higher level fusion services shall enable the automated feedback of predicted information, combined with new sensor observations, into lower level fusion processes, thereby leading to the steady reduction of forecasting uncertainties and facilitating the calibration of fusion algorithms at all levels.

Data fusion services can be structured according to their level of data and information fusion. Further, the Joint Directors of Laboratories (JDL) data fusion model is recognised as the de facto standard model with generalised data fusion levels for decision-support systems [49–51]. It provides a suitable framework for developing the future generation of reusable fusion services for web based decision-support applications in multiple domains, including the Environmental Usage Area of the Future Internet [52].

**Figure 11.**
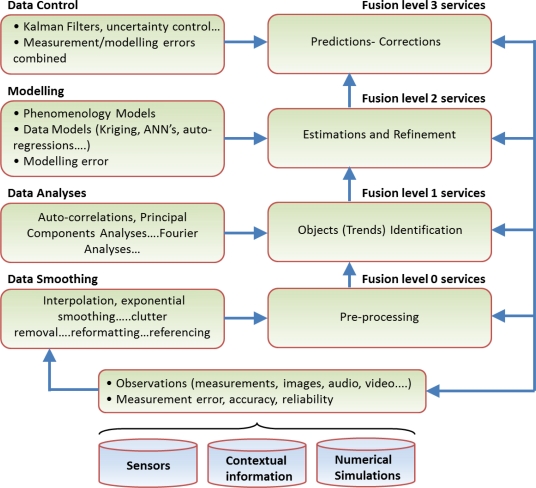
Multi-levelled Data fusion and Modelling Framework.

The JDL inspired structured data fusion and modelling framework shown in Figure 11 below is mapped against classes of data fusion algorithms. which specialise in the following:

1. Aggregation of fragmented and asynchronous data of different formats and spatial-temporal resolutions;
2. Analyses of data for discovery/detection of trends and relations and their interpretation/recognition by domain knowledge experts;
3. Modelling trends, relationships and error estimations;
4. Predicting parameters with uncertainty control through new feeds of observations, for adaptive learning and calibration of algorithms.

The uncertainty of environmental parameter predictions can be assessed depending on the expertise and reliability of human observations, on the quality and accuracy of sensor measurements and also numerical models with their respective error estimations. Fusion techniques enable the aggregation of such predictions with their weighted uncertainties using optimised filtering techniques. The fusion algorithms therefore propagate uncertainties at all levels of fusion. However, these can potentially be reduced in time and in space where the number of reliable and more accurate ground truth observations increase. These can in turn be fed into the fusion services chain with continuous feedback and adaptation of the fusion algorithms. The results of a fusion algorithm are themselves new observations in the spirit of the objective “from the Sensor to the Observation Web”. These, like all observations may be assigned a degree of belief to form the basis for decision making processes.

### 3.5. Security in Environmental Usage Area

Security-related requirements (In this paper, the word “security” indicates various aspects of data, communication and services security, authentication, authorization, privacy and trust.) of the Environmental Usage Area applications traditionally concentrated at just two issues: (i) restricting access to selected resources (products) to paying customers (Authentication and Authorization), and (ii) assuring only a very limited number of authorized persons to manipulate data and services (Confidentiality).

Internet-enabled observation systems and the real time cross-organisational observation exchange and processing have already changed this simple picture. An advanced Environmental information system should enable a wide range of use-cases for different application domains: research, decision-making, e-Government, e-Business, *etc.* Therefore it should provide support for all the main security functionalities: Authentication, Authorization (Access Control), Confidentiality, Integrity, Non-Repudiation and Auditing [53,54]. Architectural and technological solutions typically developed in the context of e-Business, or traditional Web services already exist and could be adopted as the basis for a reliable and scalable security system. However environmental applications have specific security requirements which need to be addressed.

Concerning the Authorization functionality, the diversity of possible environmental application use-cases requires the definition of a clear data policy model. Indeed, a data policy model for the environment needs to address several aspects such as the presence of different actors (users, service providers, data providers, data owners, *etc.*), the support for groups/roles (communities, virtual organizations, *etc.*), different licensing models (open licences, license approval, product ordering), and so on. In particular, it should be able to accommodate the data policies which are defined in the context of relevant initiatives, such as the ones defined by the GEOSS Data Sharing Principles [55], and by the INSPIRE Data and Service Sharing Regulation [56],

To this aim, the OGC GeoRM Working Group has developed the Geospatial Digital Rights Management Reference Model (GeoDRM RM) [57], an abstract specification for the management of digital rights in the area of geospatial data and services. In particular it defines a conceptual model for digital rights management of geospatial resources, and a metadata model for the expression of rights. The OGC also defined the GeoXACML XML dialect [58] a geo-specific extension to the OASIS standard eXtensible Access Control Markup Language (XACML) [59]. A GeoXACML-enabled access control for OGC SWE service environment has been prototyped in the SANY FP6 Integrated Project [60].

The rising importance of the wireless ad-hoc sensor networks and the development of user-centric environmental applications are leading to new classes of challenges, such as:

- How can we block “rogue” sensors from entering *ad-hoc* sensor networks on the one side, and protect user’s privacy and enforce access rights on the other side?
- How far can we “trust” the information provided by a heterogeneous group of volunteers and professionals?
- How can we assure that information is not misused, for example in order to obtain private information on users, their profiling and geolocations?
- Alternatively, how can we assure that information on state and sensitivity of the environment is not misused? For instance, public availability of observations related to whereabouts of endangered species could in some cases lead to the increase of their exposure to humans and vulnerabilities.

Although the partial solutions to the issues mentioned above have already been proposed by various authors [61–63], no comprehensive model for “secure” envirofied applications is available to this day. Some of the emerging best practices which need to be integrated in the future reference architecture for envirofied Internet applications include:

- Strict separation (for example on a Single Sign On service) of the information allowing easy identification of the user (such as name, address, e-mail...) from the application-related information contributed by the user.
- Mashing up and where needed fudging of the data related to the user’s location to assure that the user’s identity cannot be easily inferred based on his/her position. Similar precautions shall be also made for location-related information on endangered species.
- Advanced authentication and authorization mechanisms such as “smart cards” with Near Field Communication chips and NFC readers build integrated in cellular phones allowing (i) assignment of users contributions to a single contributor and (ii) upgrading the users “trust” status (for example for professional users of the system) without revealing of their identity to the service provider.
- Use of distributed quality assurance mechanisms, such as the community enabled and automatic meta-information generation currently being developed in the FP7 project TaToo [24,64] for dynamic adjustment of the observation trust status.

### 3.6. Standardization and Harmonization

Sustainability of the envirofied applications highly correlates with the price of obtaining and processing the observations. It is therefore crucial to:

1. Minimize the amount of work required for application setups and maintenance, and assure that all stakeholder groups in the Environmental Usage Area can easily add their own components for environmental monitoring (sensor systems, processing services, *etc*.); and
2. Assure that the sensors and services can be used by more than one application, across the domain and administrative borders.

This can best be achieved through open and freely available standards and harmonization of the service interfaces, protocols and data models. Sensor and sensor network technology, as well as open geospatial standards are at the very heart of the environmental domain. Organizations, such as the International Organization for Standardization (ISO), European Committee for Standardization (CEN), and the Open Geospatial Consortium (OGC), already issue related documents and define the interplay with related geospatial and common ICT technologies. The geospatial service model of ISO Technical Committee (TC) 211 and the OGC Reference Model provide two widely used cases as a foundation for environmental services. As both models are over a decade old, revision has been decided, and this provides a good opportunity to quickly influence the future foundation for the geospatial and environmental communities by including the recommended use of relevant Future Internet technologies and enablers.

The achievements from the geospatial domain are similar, but yet slightly different from common Web standards and specification languages, such as SoaML of the Object Management Group (OMG). Connections exist between geospatial standardization organizations and wider ICT-related standardization bodies (including World Wide Web Consortium (W3C) and Organization for the Advancement of Structured Information Standards (OASIS). However, mainly for historical reasons, some of their central standards are incoherent.

However standardization alone is not sufficient for two main reasons. First of all,in the environmental context, a relevant amount of sensor observations and models might be already provided as part of existing frameworks, and the providers could be unavailable to make changes. In this case the imposition of a common standard can be a limitation towards the self-growing and self-organization of the environmental observation system. Secondly, the environmental applications domain may cover use-cases not yet addressed by the standardization bodies. Therefore the need to accommodate proprietary solutions for interfaces, metadata and data models must be addressed. On the other side, especially for multidisciplinary applications, where resources conceived for different uses must be integrated, it is possible to have resources delivered according to different standards for interfaces, formats, encodings, *etc.*

This problem can be addressed resorting to harmonization solutions:

1. Avoidance of standard proliferation. The simple solution of a unique common specification for interfaces, metadata and data model is not feasible because the multiplicity of standards is not reducible over a certain extent. Multiple standards supporting similar functionalities exist and will exist in the future because they address different requirements. However standard proliferation should be avoided, adopting the relevant standards from standardization bodies or Community-of-Practices, where possible.
2. System of Systems (SoS) approach. The (SoS) concept describes the large-scale integration of many independent, self-contained systems in order to satisfy a global need [65]. It is applied in many global initiatives such as GEO for building the Global Earth Observation System-of-Systems. To maintain the component systems “independent” and “self-contained”, the SoS approach makes use of interoperability solutions at the interface level. Common standards and Special Interoperability Agreements are used only when systems need to communicate at the SoS level. The harmonization of interfaces, metadata and data models is performed by specific components in the SoS architectures.

## 4. Opportunities in the Future Internet

In Section 3, we have presented a more detailed analysis of the technical challenges encountered within the Environmental Usage Area of the Future Internet. We also provided pointers to potential solutions (“environmental enablers” for the Future Internet) and discussed the required future work. Given the capabilities and challenges outlined above, the Future Internet clearly offers the potential to advance the web of observations for multiple environmental domain sectors.

In this section, we take a more extensive look at the rising opportunities for environmental applications in the Future Internet perspective and highlight some of the opportunities for improved environmental applications, which may be deployed when the Internet of Things, Services, Content and People become integrated and ubiquitously inter-connected. This is achieved by sketching three environmental domain based scenarios, in which we expect high scientific and socio-economic impacts. Each sub-section includes a brief introduction to the environmental domain a problem statement and a description on the expected improvements made by an envirofied Future Internet. 4.1 Future Internet Enabled Biodiversity Surveys with Advanced Ontologies.

### 4.1. Future Internet Enabled Biodiversity Surveys with Advanced Ontologies

The UN Convention on Biodiversity [66] (CBD) and the EU have set a new target of halting the loss to biodiversity by the year 2020. In order to meet this goal, a solid basis for judging such progress has to be provided. Observational data on biodiversity occurrences must be merged from all available sources while assuring high quality of the resulting data sets. These should be made accessible to all interested parties.

The European Long Term Ecological Research Network (LTER-Europe) [67] maintains around 250 long term monitoring sites in 20 countries where biodiversity data is regularly surveyed together with a wealth of other parameters. These survey data are complemented with historical data and currently digitized through projects related to the Global Biodiversity Information Facility (GBIF) [68]. Nevertheless, we can greatly widen the basis from which observational data may be gleaned by additionally leveraging the potential contribution of outreach groups for data survey.

An envirofied Future Internet may modernise biodiversity data surveying by enabling humans (supported by mobile devices such as smart phones) to become one of the main contributing “sensor” for biodiversity occurrence data. It could also greatly improve our understanding of the environment and importance of the various species therein through the use of virtual and augmented reality techniques mentioned in the Section 2.3. In this sense, users contribute to the Internet of Content and People. As these human observers come from diverse backgrounds, ranging from experienced scientists to interested citizens, we must assure the accuracy of the reported observation data. In particular, the main challenge in biodiversity surveys concerns the adoption of species names and their taxonomies. These may change over time, with well-established names referred to a different species concept. An envirofied Future Internet shall overcome such problem by extending existing species ontologies with the new following concepts: (i) spatial distribution of species; (ii) temporal distribution of species; (iii) common miss-identifications and (iv) cross-referencing between species lists. These new concepts shall provide a good support to both the data quality assurance procedures for entering new survey data and also the integration of data from multiple sources in fusion processes (see also Section 3.4).

Intelligent quality assurance mechanisms can be based on knowledge of the occurrence location as well as rich species ontologies, complementing this with knowledge from users. A first level of feedback can be given to the user as a direct response to the data provision. The collected data will be merged with existing data from other sources and provided back to the users, at various levels of accuracy and aggregation depending on the user’s access rights, for further analysis. This can then be merged with data from other domains in order to enhance our understanding of local biodiversity processes but also to improve situation awareness about other relevant processes occurring at regional and global scales.

### 4.2. Future Internet Atmospheric Conditions and Pollution in “the Palm of your Hand”

Today, we have easy access to a great deal of information via television, radio and the World Wide Web. This includes pollution, pollen and meteorological data which are all relatively easily accessed in one or more dissemination channels. All this data contribute to a common sense, but they are not tailored to individual user needs. Additionally, the traditional dissemination channels often do not make use of available up to date data, and even the data available online in media is often not suited for consumers to access it directly. Further processing and contextual interpretation with situation awareness of observations and measurements are required. As the Internet of Things moves towards a sensor enabled world, even more data will become available to the users, potentially compounding the existing problem of data relevance and interpretation even more rather than assuring the users to be better informed.

The relevance and context of data are key issues for Internet users, especially those who want to receive personalised information in context with specific environmental (hazard) conditions in their local areas; for example, pollen and pollution information combined with specific atmospheric conditions. By making such data instantly available on smart phones via push and pull mechanisms, users can be not only given access to pollution and pollen data at their localised regions, from the Internet of Content, but also warned when certain parameters (or parameter combinations) exceed their own individual thresholds. Such mechanisms are already available for severe weather conditions with automatic alerts via SMS being sent to registered users based on their location. However, no systems currently exist for other types of weather and air quality conditions broadcast with impact on individual quality living. Also, none of the existing systems allow individuals to receive warnings based on their individual sensitivity to combined weather and inferred hazardous conditions.

Envirofied Future Internet applications have the potential to go beyond the state-of-the-art as they shall enable communities of users to not only personalise the data to be provided for them but also for them to provide ground truth information about meteorological, pollen and air pollutant conditions.. When it comes to atmospheric conditions and airborne hazardous materials, the users can provide three types of data:

1. Direct observations of their own environment (for example “it’s raining” or “it’s cloudy”).
2. Sensor readings. Low cost Internet enabled meteorological measurement station already exist. They can be used for this purpose, since the sensitivity of low cost gas sensors for example has recently reached the threshold where they can detect variations in urban air quality [69].
3. Subjective Observations on their response to environmental conditions (for example my eyes are itching, possibly as a consequence of excessive pollen levels).

With a secure subscription mechanism, the users shall be enabled to access, receive and provide relevant feedback information about their local atmospheric conditions. They shall also be able to define threshold limits on individual pollutants and allergens at their various spatial areas of interests in order for them to receive automated alerts from the envirofied (Future) Internet of Services. They can also contribute in bringing supporting and relevant information to other communities at large. By setting individual vulnerability thresholds, users shall receive alerts when these are exceeded, specifically according to their actual coordinate location. The data from existing monitoring networks shall be further enhanced by VGI as the users report their own observations which in turn shall feed back into the system. In addition, users shall be given the option of receiving a daily report of their actual exposure to both indoor and outdoor pollution which shall help users to develop strategies to avoid pollution concentrations, or at least limit their exposures to them.

### 4.3. Sustainable Marine Assets in the Future Internet

European marine focused technology research and innovation remains fragmented and not sufficiently market led. In line with the Innovation Union [6], the challenge for marine research and innovation is to create synergies with the market and with policy needs that are necessary to deliver significant value added to Europe from its vast marine resources. The successful implementation and development of the Integrated Maritime Policy for the European Union [70] is of great importance to the European coastal regions, as a driver of economic, social and environmental development.

For example, Ireland’s SmartOcean Innovation Strategy aims to consolidate and further develop Ireland’s current Hi-Tech Marine Services Sector. It generated €25.4 million in gross value added to the Irish economy and employed 340 individuals in 2007. Since 2007, there has been significant growth in the number of companies involved in this sector and Ireland is currently mobilising National Capabilities in ICT to capitalise on opportunities for convergence with the marine sector. These enabling technology platforms are currently deployed across a range of existing marine related sectors including shipping, security and logistics, environmental monitoring, offshore energy and emerging markets including marine renewable energy.

With the Future Internet paradigm shift, great opportunities are now up for grasp by large coastal communities in Europe [71]. They shall be able to benefit from marine environmental assets more efficiently, while protecting them and adopting new mitigation measures to those assets which have been partially degraded over time. These assets degradation are due to the lack of full understanding and awareness of environmental processes and those anthropogenic pressures put on them. This unprecedented opportunity the Future Internet is offering shall become a reality when large communities proactively contribute in marine environment observations and mass-use their existing and future sensing and mobile communication devices which are interconnected through the Internet. In an envirofied Future Internet, these communities shall be enabled using their communication devices with web applications to provide new environmental observations at localised scales and plug them into the Internet of (generic) Services such as sensor observations, web-processing and fusion and modelling.

Existing networks of *in situ* marine sensor observations with fast bandwidth communication such as in the SmartBay [72] project shall be used for capitalising on the arising Future Internet service infrastructure. New envirofied interoperable and reusable services shall be accessed to provide on demand marine environment knowledge to stakeholders at various spatial scales. This includes sea state, metocean data, water quality parameters, oil spill spatial dispersion and beaching probabilities *etc.*

With data fusion services made available (see also Section 3.4), environmental parameters shall be predicted with high spatial resolution and richer contextual and situation awareness for various stakeholder groups. These shall be also contributing in making observations which are harnessed by envirofied Future Internet services. The stakeholders are made of enabled Internet communities such as volunteer groups using social media and mobile communication networks to observe their local environment, research institutes and operating industries in coastal European regions using simulation software and measurements, for example oil spill, water quality and wave climate models, and databases of environmental parameters. Also these actions will contribute to the Internet of People, in the sense that individuals become connected and multilateral collaborations enabled.

## 5. Conclusions

This paper described the current understanding of a large European community in Environmental Informatics, including the authors, and their vision for the future implementation of Future Internet specific enablers for the Environmental Usage Area. Specifically, the authors identified a common programme for future development of generic services, bridging the gaps of existing work performed over the years by standardization bodies, mainstream service-related Internet and knowledge management groups, as well as by the owners of the first generation web based information applications in the Environmental Usage Area.

Section 1 summarized the scientific context of the presented work. Section 2 presents some recent ICT trends relevant to the Environmental Usage Area, and the Section 3 provides a more detailed analysis of the technical challenges within the Environmental Usage Area of the Future Internet. Section 3 also includes pointers to potential “environmental enablers” for the Future Internet. The enablers specialise in establishing standardized services and data encodings in which the Future Internet provides a common platform and opportunity for developing digital living labs for the Environmental Usage Area. Finally Section 4 presents an extensive outlook at the rising opportunities in the Future Internet with potentially high socio-economic impacts. This has been achieved by sketching scenarios for three environmental domains (Biodiversity surveys, atmospheric conditions and sustainable marine assets), in which we expect high scientific and socio-economic impact.

The emerging movement of the Future Internet provides an important channel to interact with in order to take a giant step forward in the advancement of existing and new information systems in the Environmental Usage Area. It also provides the environmental community existing systems the means to prepare for interfacing with the upcoming infrastructures of the Future Internet Technology Platforms. The specific requirements of the Environmental Usage Area can be directly taken into account, such that the integration work is simplified and lead to a broader and inclusive community of users coming forward. These communities will be enabled to perform open experimentations on the environmental domains and access to service infrastructures for ubiquitous web sensor observations, intelligent information and knowledge discovery, with the ability of monitoring and mitigating on environmental processes more confidently and at high resolution temporal and spatial scales. This will also enable other communities to take advantage of the envirofied Future Internet to enhance new applications in other domains with relevant environmental information and services.

## Glossary

EO: Earth Observation
FI: Future Internet
FI-PPP: Future Internet Public Private Partnership
GEOSS: Global Earth Observation System of Systems
GMES: Global Monitoring for Environment and Security
INSPIRE: Infrastructure for Spatial Information in Europe
ICSU: International Council for Science
ICT: Information and Communication Technologies
JDL: Joint Directors of Laboratory
OGC: Open Geospatial Consortium
OGC SWE, or SWE: OGC Sensor Web Enablement
SOA: Service Oriented Architecture
SEIS: Shared Environmental Information System
VGI: Volunteered Geographic Information
